# Supplementary material for: Cloning and Characterization of Drosophila melanogaster Juvenile Hormone Epoxide Hydrolases (JHEH) and Their Promoters
Source: Biomolecules. 2022 Jul 16;12(7):991. doi: 10.3390/biom12070991 (PMC9313241; doi:10.3390/biom12070991)
Supplement: Supplementary file 1 [file biomolecules-12-00991-s001.zip › biomolecules-1815543-supplementary-update.pdf]

Table S1. Primers used for sequencing *D. melanogaster* JHEH 1 cDNA

Primers	Primer sequence (5'-3')	Position	Amplicon (nt)	t_m (C^0)
DB575 (F)	ATGGGTGTCACTGTTAAAATTCT			62
DB577 (R)	GTGGAGGAAGTGTGTGCGTAACC	1-432	432	70
DB576 (F)	CTGTGGCGCGAACGCGAGGTG			77
DB579 (R)	CACAAACAAACTGGGCCAAAATT	349-864	515	65
DB578 (F)	TCGAACATGTGCAACAATTTGAG			64
DB580 (R)	CTTCGTGAGTCCTCCATCTGGCA	787-1080	293	71
DB582 (R)	GGGAACCCGCTCCAGGTGCAGGT	787-1212	425	79
DB581 (F)	GCCCTGCTGGACAACCTGATGAT			72
DB583 (R)	CTACAGAGTCTTAATTTTAAACT	1096-1411	315	54
3' RACE				
DB577 (R)	GTGGAGGAAGTGTGTGCGTAACC			70
DB265 (F)	GAGTCGGATCGACATCGT$(T)_{17}$	(-)20[a]-432	452	67
5' RACE				
DB581 (F)	GCCCTGCTGGACAACCTGATGAT			72
DB265 (R)	GAGTCGGATCGACATCGT$(T)_{17}$	1096-1470	374	67
Northern Blot Probe				
DB578	As shown above			

[a]JHEH 1 sequence (-1) to (-20) is found in supplementary materials (Figure S2). All the listed primers and directions are found in Figure 1 a. F=forward direction, R=reverse direction.

Table S2 Primers used for sequencing *D. melanogaster* JHEH 2 cDNA

Primers	Primer sequence (5'-3')	Position	Amplicon (nt)	t_m (C^0)
DB584 (F)	ATGGCGAACATCTGGCCACGAAT			72
DB586 (R)	GCTTGGCTTGGCATGAATAAAAT	1-432	432	64
DB638 (F)	GTCGGAGCTCTGACCATCCTGGTG			74
DB586 (R)	GCTTGGCTTGGCATGAATAAAAT	28-432	404	64
DB585 (F)	CCGGAGGAGTACCTCAAGAAGCT			70
DB588 (R)	CGCATACTCGCTGTCCACAAACC	349-864	515	71
DB587 (F)	AATAACACCCCTATGGGTCAGTT			65
DB590 (R)	GCCAGCCTTGGCCTTGATGGGCA	787-1215	315	78
DB589 (F)	GTGATGATCTATTATGTGACCAA			59
DB 591 (R)	TTATGAGAAATTGGCTTTCTAGA	1096-1388	292	58
3' RACE				
DB265 (F)	GAGTCGGATCGACATCGT$(T)_{17}$			67
DB586 (R)	GCTTGGCTTGGCATGAATAAAAT	(-)30[b]-462	492	64
5' RACE				
DB653 (F)	TCCATTACCACCTCCATGCGTCTGTAT	1119-1532	413	71
DB654 (F)	TCCGAATCAATGGTTGCCTCGCA	1146-1532	386	72
DB265 (R)	GAGTCGGATCGACATCGT$(T)_{17}$			67
Northern Blot Probe				
DB587	As shown above			

[b]JHEH 2 sequence (-1) to (-30) is found in supplementary materials (Figure S3). All the listed primers and directions are found in Figure 1 b. F=forward direction, R=reverse direction.

Table S3. Primers used for sequencing *D. melanogaster* JHEH 3 cDNA

Primers	Primer sequence (5'-3')	Position	Amplicon (nt)	t_m (C^0)
DB592 (F)	ATGAAGTGCCTGATAGTGTTTGG			65
DB594 (R)	TACCTTTTCGTGAATATAGTGAA	1-432	432	58
DB593 (F)	GAGCGCCAGGAGCTGTTCAACTC			73
DB596 (R)	AAAGAATCTGGACGGCAAGTACT	349-864	515	66
DB595 (F)	CTGCACACGCCACTGGCCATTCT			75
DB598 (R)	CATGGGAACCGGGGACTGCACAC	787-1212	425	75
DB597 (F)	CTGATGGTCTACTATCTGACGAA			63
DB599 (R)	TTATTTTCTCTTCTCGCCGTGTA	1096-1404	308	63
3' RACE				
DB265 (F)	GAGTCGGATCGACATCGT$(T)_{17}$			67
DB594 (R)	TACCTTTTCGTGAATATAGTGAA	(-)10[c]-432	442	58
5' RACE				
DB597 (F)	CTGATGGTCTACTATCTGACGAA	1096-1441	345	63
DB651 (F)	TCGGCCACGACGGCGGCTCGTTTT	1119-1441	322	81
DB652 (F)	TACCTGGAGAACGTGTCCAAGACG	1143-1441	298	71
DB 265 (R)	GAGTCGGATCGACATCGT$(T)_{17}$			67
Northern Blot Probe				
DB595	As shown above			

[c]JHEH 3 sequence (-1) to (-10) is found in supplementary materials (Figure S4). All the listed primers and directions are found in Figure 1 c. F=forward direction, R=reverse direction.

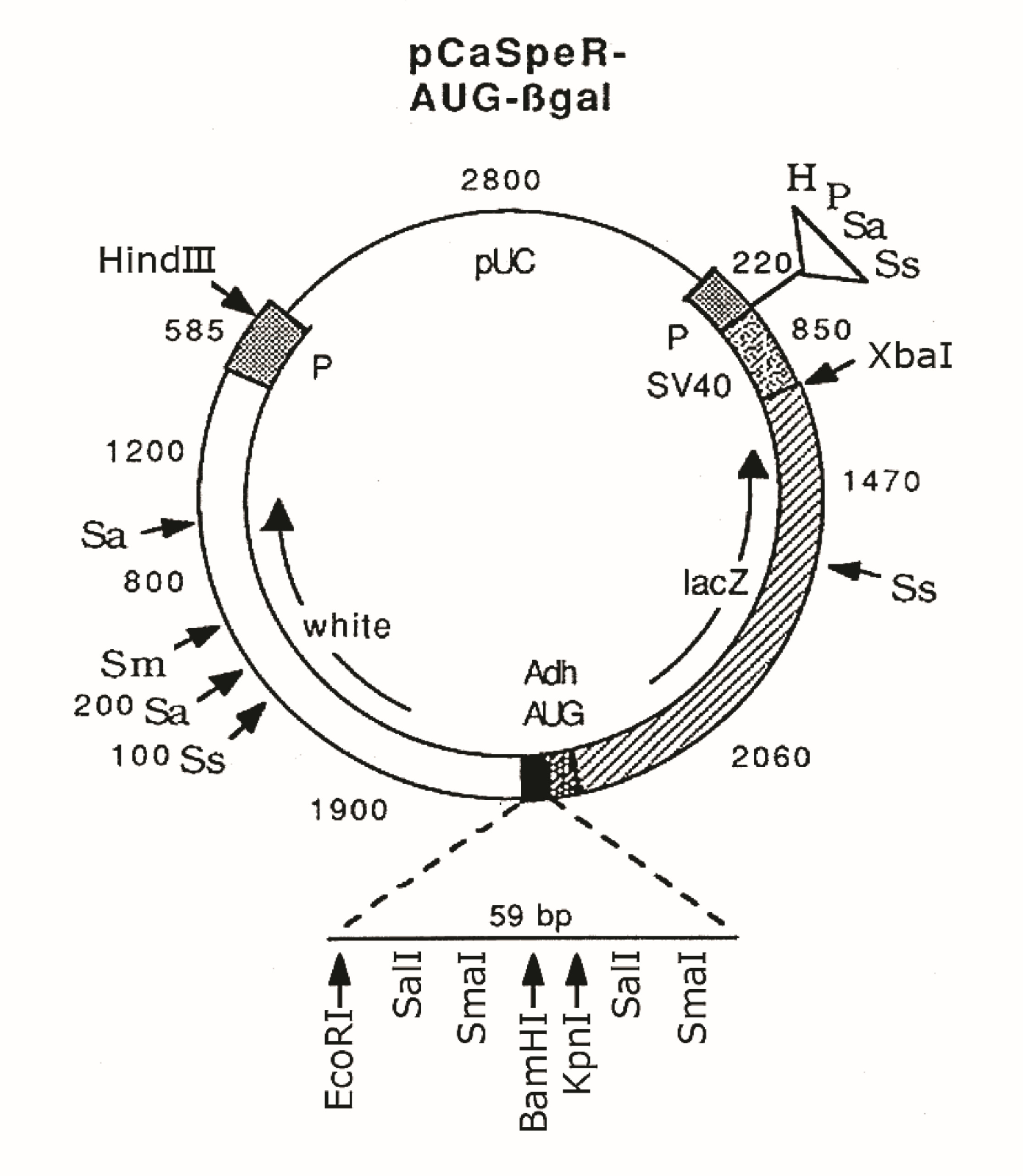

Figure S1. Plasmid pCaSpeR-AUG-bgal with unique cloning sites of *Eco*RI, *Bam*HI and *Kpn*I at the multiple cloning site denoted by arrows behind AUG start signal allowing to test promoter transcriptional activity by cloning at the multiple cloning site behind *lac*Z reporter gene. The plasmid is designed for p-element transformation of *D. melanogaster*.

Table S4. Primers used for testing *D. melanogaster* JHEH 1 promoter

Primers	Primer sequence (5'-3')	Amplicon (nt)	t_m (C^0)
DB737 (F)	CCAAGAATTCTTGTTTATCATTAGTTATAGAAGTCC		64
DB738 (R)	AATTGGATCCCTGTTCCTATCAGGCGGTTCC	846	75
DB792 (F)	CCAAGAATTCTCATAAGTATATGTACATATATGAT		61
DB738 (R)	AATTGGATCCCTGTTCCTATCAGGCGGTTCC	646	75
DB795 (F)	CCAAGAATTCTGTTCAGTCTACCCCAGTGTAGTTC		72
DB738 (R)	AATTGGATCCCTGTTCCTATCAGGCGGTTCC	446	75
DB817 (F)	CCAAGAATTCCTCCGGTCTTTACCCG		71
DB738 (R)	AATTGGATCCCTGTTCCTATCAGGCGGTTCC	305	75
DB828 (F)	CCAAGAATTCAATAAACAAACAAGCCGATT		66
DB738 (R)	AATTGGATCCCTGTTCCTATCAGGCGGTTCC	245	75
DB847 (F)	CCAAGAATTCAATAAACAAA CAAGCCGATT		66
DB738 (R)	AATTGGATCCCTGTTCCTATCAGGCGGTTCC	146	75

All forward primers (F) have an underlined *Eco*RI cleavage site and all reverse primers (R) have an underlined *Bam*HI cleavage site for cloning the different JHEH 1 promoter amplicons into pCasperR-AUG-bgal. Full sequence of JHEH 1 promoter is found in supplementary material (Figure S2). F=forward direction, R=reverse direction.

```
TTGTTTATCA TTAGTTATAG AAGTCCATTG TACATTTTCA TCGTGATTCT TGAAAGAAAC -786
DB737
GGTTATGTTT GTTGCAAAAA TTAAATCAAA TCGACTTGGT GATTGTATTT GCCTTATCTG -726
CTTTGAAAAC CGAAAGTTGA AGAGTTTTTT GTTTGGAATA CACGAGTGGC GAGCAAAAGG -666
GCAGTGTGTG GTGTTTGCTT TCATAAGTAT ATGTACATAT ATGATATATC TAAGCTCGGA -606
DB792
ACGATGTTAC GTAACTTATT CCCATGGGCT ATAGTCGACA TTGGTGTACC CTTTATAAGA -546
AATCATGTAA TATGTTGAAT TATTACTTAA CTAAATGACA GTAGAGTGAT ATTAAACCAA -486
ATGAACATCA AATTTTACTT GGCAACAAAG ATCTCACACA TGTTCAGTCT ACCCCAGTGT -426
AGTTCGAAAA TAAGGGGTAT TTTTTAGCTA ACCTGGTAGC CCAGCTGCGT CGCAAAATAT -366
DB795
ACAAAAACAA TTCGCGGGTC TCATTCTTTA TACATATAGT ACATACATAC ATACATACCC -306
CTCCGGTCTT TACCCGTAAA AGCTTCAAAC CAAAAACCGG CATGTAAGCT TTGTTGAGTA -246
DB817
AATAAACAAA CAAGCCGATT CATTCGAGTC ATCCCCGATG TGGAAAGCCG CTACTCCGTC -186
DB828
TCAGTGGAGT CGAGTCCAAA GTAAACCCGC GATAAGCCCC AACTGATCGG CGTAGGGTGG -126
DB847
TCACTCAAAG TCGCTTATCA TCATGAGCGT CGAAAACGAG AGCTGCGCCC ACGATGATAT -66
GGTAGAATCA GTGGAATCGT AGCTGCTATC CGAGACAGTC TGAGTGGAAC CGCTGATAGG -6
DB738
AACAG -1
```

Figure S2. *Jheh*1 promoter's sequence (845 bp). Horizontal arrows (pointing right) show the forwards primers and horizontal arrow (pointing left) show the back primer that were used to amplify by PCR promoter's segments that were cloned into pCaSpeR-AUG-bgal and tested for transcriptional activity.

Table S5. Primers used for testing *D. melanogaster* JHEH 2 promoter

Primers	Primer sequence (5'-3')	Amplicon (nt)	t_m (C^0)
DB787 (F)	CCAAGAATTCCATTTCAGTACCAGGGGTCATAC		71
DB740 (R)	AATTGGATCCTGTGTGTTTATGCTATATTCTTTATATATTC	1325	65
DB793 (F)	CCAAGAATTCAATTTGCACAAACTTTGGTAAGGTC		70
DB740 (R)	AATTGGATCCTGTGTGTTTATGCTATATTCTTTATATATTC	850	65
DB796 (F)	CCAAGAATTCCTTTTCGACTACCTTCTGCATAGA		70
DB740 (R)	AATTGGATCCTGTGTGTTTATGCTATATTCTTTATATATTC	585	65
DB808 (F)	CCAAGAATTCTTGACCACCTCTGTATATATTAAGG		67
DB740 (R)	AATTGGATCCTGTGTGTTTATGCTATATTCTTTATATATTC	455	65
DB848 (F)	CCAAGAATTCGCCAAAAGCTCGCCGAAATT		73
DB740 (R)	AATTGGATCCTGTGTGTTTATGCTATATTCTTTATA TATTC	245	65
DB860 (F)	CCAAGAATTCAGCTTTTGTTTGCCGGTAGC		72
DB740 (R)	AATTGGATCCTGTGTGTTTATGCTATATTCTTTATATATTC	146	65

All forward primers (F) have an underlined *Eco*RI cleavage site and all backward primers (B) have an underlined *Bam*HI cleavage site for cloning the different JHEH 2 promoter amplicons into pCasperR-AUG-bgal. Full sequence of JHEH 2 promoter is found in supplementary material (Figure S3). F=forward direction, R=reverse direction.

```
TTTATGGACT GATGAGTCTG CATTTCAGTA CCAGGGGTCA TACAGCAAGC ATTTTATGCA -1285
                                                 DB787
TTTGAAAAAT AATCAAAAGC ATTTGGCAGC CCAGCCAACC AATAGATTTG GTGGGGGCAC -1225
AGTCATGTTT TGGGGATGTC TTTCCTATTA TGGGATTCGG AGACTTGGTA CCGATAGAAG -1165
GAACTTTAAA TCAGAACGGA TACCTTCTGA TCTTAAACAA CCATGCTTTT ACGTCTGGAA -1105
ATAGACTTTT TCCAACTACT GAATGGATTC TTCAGCAGGA CAATGCTCCA TGCCATAAGG -1045
GTAGGATACC AACAAAATTT TTAAACGACC TTAATCTGGG CGGTTCTTCC GTGGCCCCCC -985
CAAAGCCCAG ACCTTAATAT CATTGAAAAC GTTTGGGCTT TTATTAAAAA CTAACGAACT -925
ATTGATAAAA ATAGAAAACG AGAGGGAGCC ATCATTGTAA TAGCGGAGAT TTGGTCCAAA -865
TTGACATTAG AATTTGCACA AACTTTGGTA AGGTCAATAC CAAAAAGACT TCAAGCAGTT -805
                                     DB793
ATTGATGCCA AAGGTGGTGT TACAAAATAT TAGTATTGTA TTTATATAAA ATAAAAAAAA -745
TTCTTATGTT GAAATTAGAT GTTAAGCTGA AATTTACTAA ATTAAGTTGA GTGAAAATAC -685
TTTTGAAGCG CAATAAACAT GTGAAAATAC TATTGACAAC TTGCATGCAT ATTTTCTTTT -625
GCTTTAAGCT TTGTACTATG AACCGTTATC TTTCGTATTT CTTTTCGACT ACCTTCTGCA -565
TAGATCAAGC TAAGCGATAA GAACTATTTC AGGCAAATCG GACAACAACA AGAAGAAATA -505
     DB796
TAACAAAAAG AAGTTGAAGT TTGCAAATAT TGTGCGTTGT GAAAATACTT TTGACCACCT -445
CTGTATATAT TAAGGGCTCC GCGTGTTGGT AATTCGAGTT CTTAATCATT ATTAATTAAT -385
               DB808
TAAATCAGTA TTTAGTTAAA TGTCATATAA CAATTTCATT TTAAGCATGA ATCGTTTCTT -325
GTTCACTTTA CTTTCGTGGA TTGATAAATG GAACTGCTTG ATCATCTTCC TAAACTAAAT -265
GTAAATTTTA AGTACAAAAA TTGCTCTCAC TCAATTTGTT GCCAAAAGCT CGCCGAAATT -205
                                   DB848
CTCAAATTAT TTGTCCAATC ATGCTCGCAT TGCATTGCCG TGTGGAATAC GATCCACTTG -145
AAATCCACAA GCCAACAAAA AGCTTTTGTT TGCCGGTAGC TTGCGCTTTA CAAATCATTT -85
                                            DB860
GTGCTCGACG ATCGTAAGCG AGGAGTTGCG CAGTTCGCCG ATCTGTGTAT ATAGAATATA -25
                                                  DB740
TAAAGAATAT AGCATAAACA CACA                                        -1
```

Figure S3. *Jheh*2 promoter's partial sequence (1325 bp). Horizontal arrows (pointing right) show the forwards primers and horizontal arrow (pointing left) show the back primer that were used to amplify by PCR promoter's segments that were cloned into pCaSpeR-AUG-bgal and tested for transcriptional activity.

Table S6. Primers used for testing *D. melanogaster* JHEH 3 promoter

Primers	Primer sequence (5'-3')	Amplicon (nt)	t_m (C^0)
DB786 (F)	GCAAAGGATCCCGTTTCCCATCATTGTTTCTGCCA		76
DB742 (R)	AATTGGATCCTGTGTGTTTATGCTATATTCTTTATATATTC	1562	65
DB794 (F)	GCAAAGGATCCGTGGCAGAAGGGCTGAGATTTATGA		77
DB742 (R)	AATTGGATCCTGTGTGTTTATGCTATATTCTTTATATATTC	852	65
DB797 (F)	GCAAAGGATCCGCAATGCACCTTGGGGCTAAGTCTA		79
DB742 (R)	AATTGGATCCTGTGTGTTTATGCTATATTCTTTATATATTC	627	65
DB809 (F)	GCAAAGGATCCGTATAAAAGTATTATAAAGTAC		63
DB742 (R)	AATTGGATCCTGTGTGTTTATGCTATATTCTTTATATATTC	452	65
DB829 (F)	GCAAAGGATCCGACCATTATAACCAAGATCA		70
DB742 (R)	AATTGGATCCTGTGTGTTTATGCTATATTCTTTATA TATTC	332	65
DB849 (F)	GCAAAGGATCCGGCGACAGCGAATTTCGATA		76
DB742 (R)	AATTGGATCCTGTGTGTTTATGCTATATTCTTTATATATTC	212	65
DB861 (F)	GCAAAGGATCCTTTATTCCGAATTCGGGTTA		70
DB742 (R)	AATTGGATCCTGTGTGTTTATGCTATATTCTTTATATATTC	112	65

All forward primers (F) have an underlined *Bam*HI cleavage site and all the backward primers (B) have an underlined *Kpn*I cleavage site for cloning the different JHEH 3 promoter amplicons into pCasperR-AUG-bgal. Full sequence of JHEH 3 promoter is found in supplementary material (Figure S4). F=forward direction, R=reverse direction.

CGTTTCCCAT CATTGTTTCT GCCACGGCGA ACCAACCACG CCCCCATTTG GCTGTCTTGC -1502
DB 786
ATGTTTGGCG GCACTTTTGT GGTTTTTTTT ATCGAGTCTC CTTGCCCCTT GAGTCCTTCG -1442
ATATAGTTGC ATGTCGACAG CATTATTGGT GCCCTTACAA TTTATGCGAA TTATCCCCTA -1382
CCTTTTATGC ACCCAGAAAT AGAAATGTGT AAAATTTAGA CAAAATATTA CCAAGTCTAT -1322
GCATATATTT GCGATGGGTT TCTATTTTAC AATTTTTATC TAATATTTTC AGTGTACAAT -1262
GTCGCCTGCT AACCTCTCGC AGTTCACTGC CTCTATTAAC TTGGCTTCAT GGTCTTCGAG -1202
CACTAACATT CTTCTACAAC ATCGACGATG TCATTATCCT CCATGGCCAA GGAGTCAAAT -1142
GTGTCCTCCT CCTGGATTTT CTCACCATCG AAGGCCAGGA TCAGAGACTC AGTAGCAACT -1082
CCAAATGCCT GGGCATATTT TTGCCTAAGA AGTGCGGCCA AAGGTTGATC CGTTCTCACA -1022
TGGCACCGCA GTTTAGGTAG ATTTGAGGAT AGAAGCCAAA TGGTTTTGCT TTGATTGGCC -962
ATTCTATTTT ACATTAGAAT TACTAGGAGT GTATTTAGAT AGTGATAGGT GCCACGTGGC -902
CTTCTGATCG GCCGACTTCA TTTCATTCAT TTTTTGGAGC CACATCATCA GTGGCAGAAG -842
GGCTGAGATT TATGATCAGT TGCCGGCGTA GCGCTTACCA ATAAATAAGT TCGATAAGAT -782
DB 794
AGTGGTGGCG AATAAGTAGC TGGGACTATT CTCAAGTCAC CATCGCACTT GTCTCATATG -722
GTACACACGT ATGGGTCACC CTATCTCGCT CGGAAATCAA TGGGCTATTC AAGGTGCAGA -662
CGCGGGCAAG TAACACAGTA CCAGGCAGAC TAACCGCAAT GCACCTTGGG GCTAAGTCTA -602
DB 797
TTATAAAATG CTGGAAATAG AAAATAATGA AAAAAAATAT ATATACTTCC TAAATACCCT -542
TTGGTTAGGG AATTGGTATT CCTAAATACC CTTAGATAGG CAAGCTATCT AACTATCTAA -482
TTATATATTA TAAGTACAAA AGTACAAAAA GTATAAAAGT ATTATAAAGT ACAAAAATTT -422
DB 809
CATTATAAAG TGCAAAGCTT TAACCACATT TAAATATATT TATACTTTAT ACTTAATTTC -362
CCAAGCCCAT TAACCTTTGA GCTGCGAGCA GACCATTATA ACCAAGATCA AGCAAAGATC -302
DB 829
AATTTGACAG ATCATATTGT GAACCACTGT GCACGATAGC CAGCGCTATC TAAACGAATT -242
TATAAATATT ATACGAACAT ATACTATAAC GGCGACAGCG AATTTCGATA GAGCTTTGCG -182
DB 849
AGACAATTCG GGGAATGAGT ATATGCGCCG GCAGTCAGTT CTTAAGCGGC TTTCTGGTTG -122
CACGGTCGTG TTTATTCCGA ATTCGGGTTA GGAATCACAA ACCATTATCT CTTCTGGGAG -62
DB 861
GTGTACGAGG TGGCTAATTT AAATTCATAA CGAGGGGGAG CTAATTGCGC CTGTGGCCAG -2
DB 742
-1

A

Figure S4. *Jheh*3 promoter's sequence (1562 bp). Horizontal arrows (pointing right) show the forwards primers and horizontal arrow (pointing left) show the back primer that were used to amplify by PCR promoter's segments that were cloned into pCaSpeR-AUG-bgal and tested for transcriptional activity.

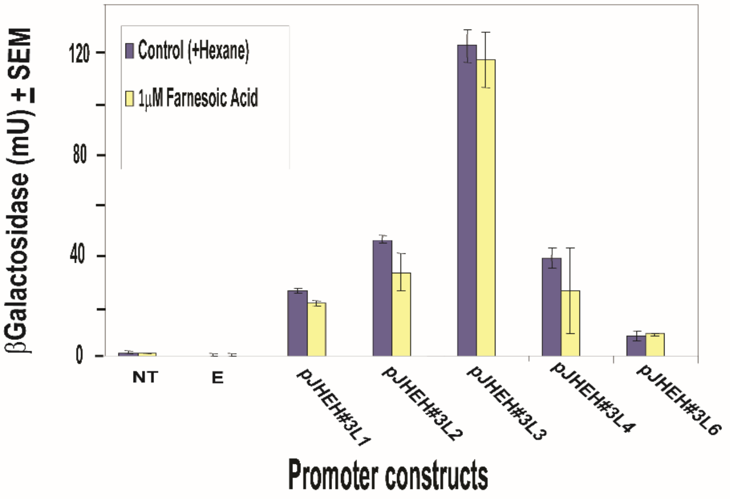

Figure S5. Effect of farnesoic acid 1 mM on transcriptional activity of transformed D.Mel2 cells with different length *jheh*3 promoter constructs (yellow bars). Controls were treated with hexane (magenta bars). Non transformed cells (NT), cells transformed with empty plasmid (E). See Figure 7 for promoter's sequence lengths.
